# Regulation of Innate Immune Responses by Platelets

**DOI:** 10.3389/fimmu.2019.01320

**Published:** 2019-06-11

**Authors:** Lucas Secchim Ribeiro, Laura Migliari Branco, Bernardo S. Franklin

**Affiliations:** ^1^Institute of Innate Immunity, University Hospitals, University of Bonn, Bonn, Germany; ^2^Centro de Terapia Celular e Molecular (CTC-Mol), Universidade Federal de São Paulo, São Paulo, Brazil

**Keywords:** innate immunity, platelets, inflammation, leukocyte migration, cytokine production, cell survival

## Abstract

The role of platelets has been extensively studied in the context of coagulation and vascular integrity. Their hemostatic imbalance can lead to known conditions as atherosclerotic plaques, thrombosis, and ischemia. Nevertheless, the knowledge regarding the regulation of different cell types by platelets has been growing exponentially in the past years. Among these biological systems, the innate immune response is remarkably affected by the crosstalk with platelets. This interaction can come from the formation of platelet-leukocyte aggregates, signaling by direct contact between membrane surface molecules or by the stimulation of immune cells by soluble factors and active microparticles secreted by platelets. These ubiquitous blood components are able to sense and react to danger signals, guiding leukocytes to an injury site and providing a scaffold for the formation of extracellular traps for efficient microbial killing and clearance. Using several different mechanisms, platelets have an important task as they regulate the release of different cytokines and chemokines upon sterile or infectious damage, the expression of cell markers and regulation of cell death and survival. Therefore, platelets are more than clotting agents, but critical players within the fine inflammatory equilibrium for the host. In this review, we present pointers to a better understanding about how platelets control and modulate innate immune cells, as well as a summary of the outcome of this interaction, providing an important step for therapeutic opportunities and guidance for future research on infectious and autoimmune diseases.

## Introduction

Platelets are small disc-shaped cells derived from the fragmentation of megakaryocytes (MKs), in a process regulated by the binding of thrombopoietin (TPO) to its receptor (1–3). The human body contain around 750 billion circulating platelets and it is able to generate 200 billion new cells per day from its precursors in the bone marrow (4, 5) and in the lungs, as recently described (6). These cells stay in circulation for up to 10 days and they are later captured in the liver and in the spleen for degradation. As they grow senile, platelets lose their membrane sialic acid residues and reduce the TPO incorporation, an indication for their clearance. The decay is sensed by Ashwell-Morell receptors and, in a JAK2/STAT3-dependent mechanism, stimulate hepatocytes for the production of TPO, in order to command the generation of new platelets (7, 8). Since they are basically organized pieces of cytoplasm, platelets carry over several MK-derived molecules and factors that can be released upon activation. They do not have a nucleus, but are rich in mitochondrial DNA and RNA, the latest being useful for *de novo* protein synthesis (9–13). Small molecules, nucleic acids, lipid mediators and proteins can be stored in different types of organelles: alpha-granules, dense granules and lysosomal vesicles (14–16). Once activated, platelets undergo drastic shape changes and can release these factors to the extracellular compartment in their soluble forms or enclosed within bioactive microvesicles (17–19). Most of these secreted components and the membrane-bound proteins present in the vesicles have an important role in the control of the immune system. Given the ubiquitous nature of this featured cell type and the growing interest in its part in the defense of the organism (20, 21), here we present a brief summary of some of the effects that platelets can exert over innate immune cells, especially neutrophils, monocytes and macrophages.

## Platelet Roles in Immune Cell Migration, Phagocytosis and Pathogen Clearance

**Figure 1:**
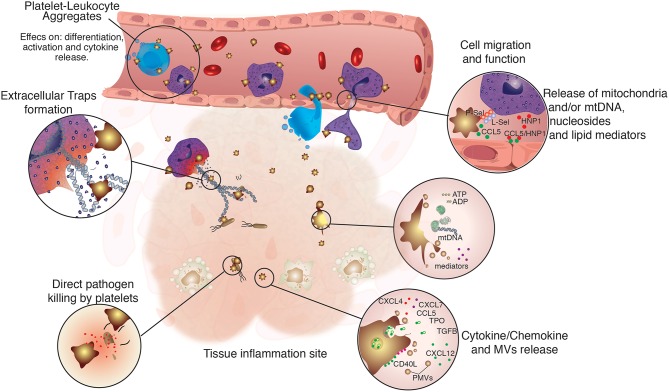
Regulation of immune cell function by platelets. Platelets are not only directly involved in immune defense, but also assist and regulate several functions of innate immune cells. Platelets were shown to participate directly in the modulation of immune cells function by physically tethering to them, or by the release of platelet-derived microvesicles (PMVs), lipid mediators, nucleosides and mitochondrial DNA, growth factors, as well as cytokines and chemokines. Platelets and their releasates have broader effects on the differentiation (29–32), migration (33–35), phagocytic, and microbicidal activities (36–38), formation of extracellular traps (39–41), pathogen clearance, and cytokine response of innate immune cells (42–45).

Besides their known functions in hemostasis, platelets can also play an important role in the body defense against invading pathogens. They have a plethora of membrane receptors able to detect pathogen- and danger-associated molecular patterns (PAMPs and DAMPs), such as Toll-like receptors (TLRs) (22–26). Hence, platelets represent a prompt source of immune mediators, secreting several factors that act both on the invading pathogen or on surrounding cells (27, 28) (Figure 1).

Beyond the active function as immune cells, platelets guide leukocytes to injury site, enhancing and facilitating their immune functions. In the liver, platelets transiently scan Kupffer cells through interactions between the von Willebrand factor (VWF) and its receptor on platelets (glycoprotein Ib alpha chain gpIbA, also known as CD42b). Upon signs of infection, platelets are triggered by the Kupffer cells via the integrin GPIIb (CD41) to promote stronger adhesion, and to enclose microbes, facilitating their clearance. Mice with inactive platelet receptors display increased inflammation markers and higher mortality upon infection (46). In the blood, platelets contribute to microbial clearance and priming of adaptive responses by redirecting blood-borne bacteria to splenic dendritic cells (DCs) in a manner dependent on GPIb and complement C3 (47).

Apart from the direct clustering with microbes, platelets also contribute to pathogen clearance by coordinating the activity of immune cells such as neutrophils, which probe activated platelets via the P-selectin glycoprotein ligand-1 (PSGL-1, CD162) in order to trans-migrate to inflammatory sites. In platelet-depleted mice, or when the PSGL-1 interaction with its receptor on neutrophils was blocked, the neutrophil typical crawling was suppressed (42). These results were further validated by a novel *ex vivo* microfluidic system that allowed a better understanding of the interaction between these two cells types and the importance of the P-selectin and PSGL-1 (P-selectin glycoprotein ligand-1) in that context (48). Curiously, this same interaction also leads to the generation of neutrophil-derived vesicles filled with arachidonic acid, which are promptly internalized by platelets via Mac-1. Once inside, the arachidonic acid can be converted into thromboxane A_2_ (TXA_2_). Next, the platelet-derived TXA_2_ acts on the neutrophil, increasing the expression of ICAM-1 and consequent crawling and extravasation (43).

Depletion of platelets in a murine model of sepsis reduced the edema and neutrophil influx to the lungs and bronchoalveolar compartment, through the suppression of Mac-1 expression in the neutrophils (49). It has been also demonstrated that serotonin (5-HT) plays a major role in neutrophil adhesion and rolling. As platelets are the major source of peripheral serotonin, pharmacological and genetic inhibition of 5-HT signaling resulted in significant reduction in cell rolling and extravasation to lungs, peritoneum and skin wounds, as well as increased survival under LPS-induced shock, supporting the action of platelet-derived 5-HT on innate immune cells (50).

In case of breached endothelium, platelets can be recruited via CD41 and CD42b and cover a large area around the damage, proceeding to cover—but not occlude—the blood vessel. Migrating neutrophils then use the platelet scaffold to attach and crawl toward the inflammation site (33). It was also shown that this initial interaction between platelets and neutrophils can also bring inflammatory monocytes to the site of damage, in a CD40-CD40L-dependent mechanism. The blockage of this signaling cascade leads to inefficient migration, uncovering the critical role of platelets for diapedesis (34). Platelets also associate and synergize with neutrophils to promote monocyte recruitment through heteromers of platelet-derived CCL5 and neutrophil-derived HNP1 (alpha-defensin), which mediate monocyte adhesion through CCR5. Disruption of HNP1-CCL5 interactions attenuated monocyte and macrophage recruitment in a mouse model of myocardial infarction (35). Also, when platelets are activated, they release the content of their alpha granules, exposing important mediators such as adenosine diphosphate (ADP). The binding of this molecule to P2Y receptors (51) leads to quick translocation of P-selectin to the membrane, increasing the potential for recruitment of neutrophils, monocytes and lymphocytes to the injury site (52). Once they are recruited, the activity of leukocytes seems to be also dependent on ADP: the chemical blockage of the P2Y12 receptor results in diminished production of reactive oxygen species (ROS) by mouse and human neutrophils (53). Platelet-released HMGB1—a critical protein for the onset of thrombosis (54)—has also been involved in the recruitment and survival of immune cells (55). Mice lacking this protein in platelets showed lower monocyte migration to the inflamed tissue in a mechanism dependent on the receptor for advanced glycation end products (RAGE) and TLR4-derived apoptosis (56). Recent *in vitro* data also point that CXCL4 (also known as PF4, platelet factor 4)—an important chemokine secreted by activated platelets—might be involved in monocyte migration upon binding to CCR1 receptor (57). The molecular pathway involving the platelets on leukocyte recruitment can range from adhesion, crawling, diapedesis and tissue invasion to injury clearance and inflammation resolution and the mechanisms observed should be further studied and explored (58). Those concepts are summarized in Figure 1.

Platelets can not only drive cells to an inflamed site, but also actively move in the direction of the injury. Using a *in vivo* platelet-reporter model, it was shown that mobile platelets are capable of active adhesion and rolling by interacting with the endothelium, in a process dependent on ADP and TXA_2_. Moreover, platelets can wrap and collect invading bacteria, acting as scavengers and enhancing the activity of phagocytes, such as neutrophils (5).

The formation of leukocyte-platelet aggregates also constitutes a hallmark in the modulation of innate immune cells by platelets. Bacteria can activate platelets by increasing the potential interaction with neutrophils, leading to enhanced phagocytosis, killing and clearance, in a mechanism dependent on TLR recognition (59). A cell-conditional model has shown that mice submitted to platelet depletion were prone to bacteremia to *Staphylococcus aureus* (60). It has also been reported that thrombin-activated platelets, as well as their releasates, can increase the engulfing and extermination of Gram-positive bacteria in bone marrow-derived dendritic cells, macrophages, and neutrophils. In the two first cell types, the effect was dependent on cytoskeleton remodeling. In DCs, the binding of CD40-CD40L was critical for the inflammatory response. In macrophages, platelets also play an important role in the restriction of *S. aureus* infection (36, 37, 61). Besides blood-borne bacteria (47), platelets can also form aggregates with erythrocytes infected with *Plasmodium*, leading to the killing of the parasite. The platelet count, erythrocyte-platelet complexes and platelet-associated killing were inversely correlated with parasite loads, suggesting that platelets may contribute to the pathogenesis and control of the human malaria parasite (38, 62).

In addition to the platelets themselves, platelet-derived microvesicles (PMVs) play an important part in their communication with endothelium and innate immune cells. PMVs are the most abundant circulating particles in the body and can be loaded with nucleic acids, proteins, lipids, and small molecules originated from the platelets or MKs. Using a molecular approach to this system, it was shown that PMVs loaded with the microRNAs could modulate the transcription of different mRNA in macrophages, reprograming them toward a phagocytic phenotype (63). Interestingly, besides stimulating more effective pathogen uptake by leukocytes, platelets also have phagocytic activity reported in several different models (64).

## Regulation of Immune Cells Function

Cytokines and chemokines are fundamental pieces in the origin, growth, differentiation and function of immune cells. They compose a consistent and tunable communication channel that aims to keep the organism in a state of homeostasis. Injuries, infections, and autoimmune reactions interrupt this balance, leading to the production of massive amounts of such highly reactive components (65). Since platelets are so widely distributed in the body, it is reasonable to assume that they can sense these fluctuations and react properly in order to return to the homeostatic status, by direct contact or secretion of soluble factors, such as CCL5, CXCL4 and CD40L.

It is known that the adhesion of monocytes to platelets—a common event under inflammatory conditions—will result in the translocation of NFκB to the nucleus, where it will trigger the enhance the expression of CCL2 and IL-8 by monocytes. A second signal from the platelet, CCL5, will activate the release of the monocyte pro-inflammatory chemokines and reinforce the interaction via P-selectin (66). Also the chemokine CXCL4 has an important role on systemic inflammation, such as in septic lung injury. Upon an inflammatory injury, platelets release CXCL4 via Rac-1, promoting neutrophil recruitment, edema, tissue damage, and high levels of CCL5, CXCL1, and CXCL2. The pharmacological neutralization of CXCL4 reduced the levels of pro-inflammatory factors and improved the overall condition of the animals (67, 68). Platelets are cellular sources of CD40L (CD154) and this protein has a strong effect on leukocytes. In systemic lupus erythematosus (SLE), platelets were found to be activated by immune complexes formed between autoantibodies via FcγRIIA and then driven to the formation of aggregates with monocytes and plasmocytoid DCs. The consequence of this interaction was an increased IFN-α release by the latter cells via CD40/CD40L. In the same study, an experimental murine model showed that the depletion or blocking of platelets in lupus-susceptible mice evoked better clinical parameters, while platelet transfusion aggravated the disease (44). The interaction of neutrophils and platelets via CD40 is also known to activate a positive feedback loop characterized by the increased release of superoxide and reactive oxygen species by the leukocytes, stimulating the secretion of CD40L by platelets (69). In the presence of autologous platelets, monocytes from older donors have greater capability for production of IL-8 and CCL2, when compared to young adults, in a mechanism initiated by granzyme A secreted from platelets. The inhibition of this factor restored the levels of IL-8 and CCL2 in a TLR-4/Caspase-1 dependent manner. However, the classical markers of platelet activation– P-selectin, CCL5, and CXCL4—were not correlated with this effect (70).

An important and tightly regulated cytokine deserves attention: IL-1β is the product of a pre-protein cleaved by caspases, as one of the outcomes of inflammasome activation (71, 72). Leukocytes are a major source of this cytokine in the body and given the constant interaction among these cells, one can speculate how platelets can modulate the expression of this critical protein. In a cohort consisted of 500 healthy individuals, a correlation was found between platelets and plasma levels of IL-1β, in different scenarios: there is a positive association of platelet counts and the plasmatic concentration of the cytokine and the expression of P-selectin was linked to higher levels of IL-1β and IL-6 after *ex vivo* stimulation (73). Looking deeper into mechanisms for such event, it was reported that, after a viral infection, platelets would release PMVs filled with the IL-1β, as a result of to NLRP3 activation by reactive oxygen species (74). Next, healthy monocytes exposed to platelets from infected patients secreted more cytokines, such as IL-1β, IL-8, IL-10, and the chemokine CCL2. This effect required the formation of platelet-monocyte aggregates (PMAs), but it was not present when healthy platelets were used (75). Other studies indicate that platelets themselves could be a source of inflammasome components, including IL-1β itself and also IL-18, another inflammasome-related cytokine (76–81). Nevertheless, the presence of some IL-1 cytokines on platelets is disputed. Part of the discrepancies may be due to the process of platelet isolation from fresh whole blood, which is laborious and prone to contamination by leukocytes. Given the capacity of reaction by these nucleated cells, the smallest proportion (1:10^5^) of leukocytes within in a platelet suspension can lead to misleading interpretations (82). Therefore, further studies are necessary in order to establish the role of platelets as source of IL-1 cytokines and on their effects on the IL-1β production by immune cells.

Despite abundant reports of pro-inflammatory effects of platelets on innate immune cells, platelets were also shown to dampen inflammation by direct interaction or release of different factors. A critical importance of platelets in the modulation of the immune response in sepsis was recently shown. Using a mouse model of platelet depletion, by chemical or genetic intervention, it was reported that platelets and their releasates can reduce the concentration of pro-inflammatory cytokines such as TNF- α and IL-6 after sterile or infectious stimuli. Platelet depletion led to greater mortality and organ failure in a mouse model of septic shock, while administration of platelets dampens the generalized and detrimental immune response. Platelets protected against septic shock through the activation of the COX-1-PGE_2_-EP_4_ pathway on macrophages (45). Other independent studies showed that the addition of platelets or their supernatants to a culture of mononuclear cells led to suppressed production of IL-6 and TNF-α and higher production of IL-10 after stimulation with PAMPs from different origins (83). The blockage of CD40-CD40L prevented the modulating effects, demonstrating the importance of the duo signaling for the platelet regulatory effect (84). Later, similar effects on TNF-α and IL-10 secretion by macrophages and monocytes were also found to be related to the release of PGE_2_ by platelets and its binding to specific prostanoid receptors (85). Soluble factors secreted by platelets are also able to regulate the expression of mRNA of inflammatory markers, including reduced levels of NOS2 (iNOS) and consequent suppressed production of nitric oxide (NO), followed by inhibition of NFκB signaling and higher arginase-1 expression (86, 87). A clinical approach was used to test whether platelet concentrates used in blood transfusion would have an effect on the response of dendritic cells. Upon mimicking of viral and bacterial infection, the myeloid cells showed a reduction in co-stimulatory molecules and reduced production of IL-6, IL-8, IL-12, IP-10, and IFN-γ, suggesting that the patients undergoing platelet transfusion might not be able to assemble proper response against infectious threats (88).

## Interaction With Leukocytes for the Formation of Extracellular Traps

Neutrophil extracellular traps (NETs) are physical barriers composed by cytoplasmatic proteins and nuclear content, expelled to the extracellular compartment in order to capture and eliminate pathogens, especially in systemic inflammation (89). Shortly after the original description, it was shown that platelets play a fundamental part in the formation of these structures in a septic model, by initially detecting TLR4 ligands and inducing adhesion to neutrophils (39). Circulating bacteria can be entangled by these structures, especially in the liver sinusoids and lungs capillaries, stopping them from disseminating through the bloodstream. Later, it was reported that the mechanism involving the bond between platelets and neutrophils was dependent on α_L_β_2_-integrin LFA-1 (CD11a/CD18) (40).

Besides the known activity in inflammation and other infectious diseases (90, 91), the role of platelets and NETs in systemic sclerosis was recently discovered. PMVs loaded with the protein HMGB1 are abundant in patients and in mouse disease models, were able to cause the formation of NETs, with higher proteolytic activity and degranulation (41, 55, 92). The interaction of platelet GPIb with neutrophil CD18 plus the release of VWF and CXCL4 are involved in the formation of NETs. This event is dependent on the production of thromboxane A2 and can be inhibited by aspirin and prostacyclin, showing the importance of platelet components for the NET formation (93, 94). On top of the formation of the extracellular traps, activated platelets can also trigger other inflammatory processes. In a model of venous thrombosis, platelets were shown to induce neutrophil death by necroptosis, via MLKL and RIPK1, leading to cell aggregation and final clot formation (95). Macrophages can also form extracellular traps as shown in a model of acute kidney injury. In the event of rhabdomyolysis derived from muscle damage, platelets are activated by the heme group released by the necrotic muscle and serve as scaffold for the formation of the macrophage extracellular traps (96).

## Changes in Cell Markers and Consequences to Innate Immunity

Platelets are able to modulate other functions of innate immune cells trough the release of inflammatory mediators or through cell-cell contact (97). This interaction can lead to changes in cell markers and phenotype, induce mutual cell activation and cytokine production that are implicated in the pathogenesis of inflammatory diseases and in the resolution of infections. Monocytes are central hubs of the innate immune system that present high plasticity and possess both pro-inflammatory and anti-inflammatory properties and can also maturate into macrophages and dendritic cells. Human monocytes can be divided in three different subsets accordingly to the expression of CD14 and CD16: classical monocytes (CD14^high^CD16^−^), intermediate (CD14^high^CD16^high^) and non-classical monocytes (CD14^low^CD16^+^) (98). Even though the functions of different subtypes of monocytes are still controversial and context-dependent, CD16^+^ monocyte subsets are related with inflammatory features, such as the release of IL-1β and TNF-α, the differential expression of TLRs, scavenger receptors and the expression of the co-stimulatory molecules CD80 and CD86 (99).

The CD16^+^ monocyte subsets are also associated with the PMAs. These complexes are linked with various inflammatory diseases, such as acute thrombotic events, diabetes and auto-inflammatory disorders and are markers for both platelet and monocyte activation (97). The co-incubation of platelets with monocytes to induce PMAS formation lead to the shift of CD14^high^CD16^−^ monocytes toward to the CD14^high^CD16^+^ subtype. The physical interaction of activated platelets and monocytes is mainly mediated by P-selectin-PSGL-1 and induce up-regulation of COX-2, which induce an higher expression of both integrins CD11b and CD11c (97). Moreover, it was reported that PSGL-1 engagement also increased the expression of the integrin CD49d (α_4_β_1_) and decreased CD62L expression. The phenotypic changes promoted by platelet interaction boosted monocyte adherence to the activated endothelium through a higher binding to fibronectin, vascular cell adhesion protein 1 (VCAM-1) and intercellular adhesion molecule 1 (ICAM-1) (100). It was also described that PMAS formation in rheumatoid arthritis drive the induction of the pro-inflammatory CD14^high^CD16^+^ monocyte subset via the increased expression of CD147 on activated platelets (101).

In serum-free conditions cytosolic fractions of platelets were able to induce higher expression of CD16 and carboxypeptidases, reinforcing the importance of cell-cell contact to induce monocyte maturation (29). However, even though cell-cell contact might be critical to the regulation of monocytes, it was reported that the local release of TGF-β by activated platelets also leads to the expression of CD16 on infiltrating or resident monocytes, facilitating the lysis of murine anti-CD16 hybridomas (29). Platelets also contribute to the generation of CD14^+^CD16^+^ dendritic-like cells (DLCs) from peripheral blood monocytes. Cultivation of purified CD14^+^ monocytes with immobilized P-selectin in the presence of M-CSF and IL-4 induced the differentiation into CD14^+^CD16^+^ DLCs with increased expression of CD1a. The resulting DLCs presented reduced phagocytic activity and increased alloreactivity to naive T cells. Interestingly, P-selectin interaction with monocytes was also able to inhibit monocyte differentiation into macrophage in response to M-CSF (102).

Monocytes and platelets are fundamental parts in several inflammatory diseases and one of the most important conditions is atherosclerosis, a chronic inflammatory disease characterized by the formation of plaques in the arteries resulting from lipid accumulation and inflammation (103). Platelets are important players for the genesis and progression of the disorder due to their ability to interact with immune and endothelial cells and through the uptake of low-density lipoproteins (LDL). The release of soluble inflammatory mediators such as CCL5, CXCL4, and CXCL7 by platelets induce the migration and activation of monocytes, dendritic cells and neutrophils to the damaged site, contributing to the progression of atherosclerosis (104). Platelets also secrete CXCL12 that mediate the chemotaxis of CD34^+^ progenitors to the sites of injury and promotes their differentiation into endothelial and macrophages/foam cell phenotype (30, 31). In mice, the dual engagement of CXCR4 and CXCR7 by platelet-derived CXCL12 induced the differentiation of monocytes into CD163^+^ macrophages, that contributes to hemoglobin clearance and thus it was associated with atheroprotection. However, it was described that CD163^+^ macrophages were associated with plaque progression, microvascularity, and up-regulation of hypoxia-inducible factor 1α (HIF1α) and vascular endothelial growth factor A (VEGF-A) in human atherosclerotic lesions samples, suggesting that these cells can also exert a pro-inflammatory role (32).

Monocytes that migrate to the atherosclerotic lesions can further differentiate into macrophages foam cells that upregulate the scavenger receptor CD36. This receptor recognize pathogens and apoptotic cells but also oxidized LDL (oxLDL) (105). The local release of CXCL4 promotes monocyte maturation into macrophages and support the retention of LDL on cell surfaces (106). Also, the uptake of oxLDL by macrophages is boosted by PF4, CLXC4, CXL12, and platelet-derived growth factor (PDGF) (32). Moreover, platelet uptake of oxLDL induce platelet apoptosis and facilitates its phagocytosis by monocytes and macrophages, also contributing to foam cell formation (32). Even though monocytes and macrophages are the key players in atherogenesis, neutrophils, and platelet-neutrophil aggregates (PNA) also have an important role in this process (107). Similarly to what is observed in PMAs, P-selectin-PSGL1 interaction is crucial to the formation of PNAs and induce an higher expression of CD11b/CD18 on neutrophils, contributing to their activation and adherence to the activated endothelium (108).

## Role on Cell Death and Survival

Apoptosis is a programmed cell death that contribute to terminate immune responses and control inflammation (109). Although the precise mechanism by which platelets can prevent human polymorphonuclear (PMN) leukocyte and monocyte cell death is still not established, there are evidence in the literature suggesting that platelets can increase cell survival.

Co-cultivation of neutrophils with thrombin-treated or untreated platelets was able to reduce neutrophil apoptosis in comparison with neutrophils cultured alone, in a mechanism that seems to be independent of P-selectin (110). In another study, TGF-β derived from thrombin-treated platelets or exogenous TGF-β was able to reduce neutrophil apoptosis in a dose-dependent manner (111). In atherosclerosis, the release of CXCL4 was reported to prevent neutrophil and monocyte apoptosis (104). Adenosine 5′-diphosphate-activated platelets co-cultivation with isolated neutrophils from patients with acute coronary syndromes was also able to reduce neutrophil cell death (112). Also, the uptake of platelets by monocytes downregulates caspase-9 and caspase-3, suppressing monocyte apoptosis (113).

Platelets can also favor cell survival trough the induction of autophagy on neutrophils. Autophagy is a highly conserved biological process responsible for the degradation of organelles and cellular components. This pathway can be activated in response to starvation in order to replenish nutrient stores or to avoid the generation of toxic byproducts derived from unwanted organelles and proteins, contributing to maintain cellular homeostasis (114). HMGB1 released by activated platelets in coronary thrombi and its binding to RAGE receptors expressed on neutrophils was shown to promote the autophagic pathway on these cells. The induction of autophagy prevented apoptosis and enhanced cell survival, priming neutrophils for NET generation and contributing for venous thrombosis (92). Moreover, autophagy-dependent NET formation was also described to contribute to lung fibrosis (115). Since it was already described the role of platelets in this condition (116), these cells might also contribute to induce the autophagy-dependent NET in fibrosis.

The ability of platelets to induce pro-survival signaling can contribute to innate immune cell functions but also contribute to exacerbate the inflammation in different diseases. Since apoptosis induction is fundamental to ensure the resolution of the inflammatory response, the pharmacological modulation of cell death can have beneficial effects in inflammatory diseases (117).

## Perspectives

Previously known as keepers of hemostasis, platelets gained importance over the last years due to exciting discoveries that place them as critical players of the innate immune system. Platelets can contribute to the resolution of infections and the genesis and progression of autoimmune and inflammatory diseases directly or through the regulation of immune cells. More recently, there is a growing interest and evidence in the literature suggesting that platelets can also be a target to treat inflammatory conditions with promising results. However, further studies are necessary to better understand how platelets modulate the immune response. The deep comprehension of platelet role in infections and diseases will permit the development of therapeutic strategies to treat conditions in which platelets have a detrimental role.

## Author Contributions

All authors have read and approved the publication of this manuscript. LSR and LM wrote the manuscript with input from BF.

### Conflict of Interest Statement

The authors declare that the research was conducted in the absence of any commercial or financial relationships that could be construed as a potential conflict of interest.
